# Viral and Bacterial Interactions in the Upper Respiratory Tract

**DOI:** 10.1371/journal.ppat.1003057

**Published:** 2013-01-10

**Authors:** Astrid A. T. M. Bosch, Giske Biesbroek, Krzysztof Trzcinski, Elisabeth A. M. Sanders, Debby Bogaert

**Affiliations:** Department of Pediatric Immunology and Infectious Diseases, University Medical Center-Wilhelmina Children's Hospital, Utrecht, The Netherlands; University of Alberta, Canada

## Abstract

Respiratory infectious diseases are mainly caused by viruses or bacteria that often interact with one another. Although their presence is a prerequisite for subsequent infections, viruses and bacteria may be present in the nasopharynx without causing any respiratory symptoms. The upper respiratory tract hosts a vast range of commensals and potential pathogenic bacteria, which form a complex microbial community. This community is assumed to be constantly subject to synergistic and competitive interspecies interactions. Disturbances in the equilibrium, for instance due to the acquisition of new bacteria or viruses, may lead to overgrowth and invasion. A better understanding of the dynamics between commensals and pathogens in the upper respiratory tract may provide better insight into the pathogenesis of respiratory diseases. Here we review the current knowledge regarding specific bacterial–bacterial and viral–bacterial interactions that occur in the upper respiratory niche, and discuss mechanisms by which these interactions might be mediated. Finally, we propose a theoretical model to summarize and illustrate these mechanisms.

## Introduction

### Colonization as a Crucial Step in the Pathogenesis of Respiratory Disease

Acute respiratory infections, in particular pneumonia, remain one of the most important causes of death in both adults and children, with an estimated 3.5 million deaths worldwide in 2008. Sharp peaks in mortality due to respiratory infections are observed during infancy and late adulthood. With approximately 1.4–1.8 million fatal cases per year in children under the age of five, pneumonia causes more fatalities than AIDS, malaria, and measles combined [1], [2]. Although pneumonia is the most important cause of death, acute middle ear infections also cause a major burden to global health. At the age of three years, up to 80% of children have suffered at least one episode of acute otitis media, while more than 40% have experienced more than six recurrences by the age of seven, even in high-income countries [3]. Associated sequelae and direct and indirect costs have important socioeconomic consequences for public health care.

The human upper respiratory tract is the reservoir of a diverse community of commensals and potential pathogens (pathobionts), including *Streptococcus pneumoniae* (pneumococcus), *Haemophilus influenzae*, *Moraxella catarrhalis*, and *Staphylococcus aureus* [4], which occasionally turn into pathogens causing infectious diseases. To cause respiratory disease, bacteria first need to colonize the nasopharyngeal niche. Colonization of this niche is a dynamic process: acquisition and elimination of species, interactions among microbes and between microbes and the host, and interference by environmental factors are suggested to cause a dynamic and complex microbial interplay. In a balanced state, this ecosystem as a part of the complete human microbiome is assumed to play a major beneficial role for the human host [5]. However, imbalances in this respiratory microbial community can contribute to acquisition of a new bacterial or viral pathogen, carriage of multiple potential pathogenic bacteria, or a viral co-infection [6]. Subsequently, imbalances in the ecosystem may result in overgrowth and invasion by bacterial pathogens, causing respiratory or invasive diseases, especially in children with an immature immune system.

The focus of this review is to describe current knowledge on microbial interactions between commonly detected bacterial and viral pathogens in the upper respiratory tract, with a focus on the mechanisms by which these interactions are potentially mediated. We will conclude by incorporating the presented information into a single theoretical model of interplay between viral and bacterial species, which we believe to be a crucial first step in the pathogenesis of respiratory and invasive diseases.

## Bacterial Interactions

In 1960, Hardin [7] stated that completely competitive species cannot colonize the same ecological niche, indicating that one microorganism has the possibility of fully extinguishing another. However, the concept of colonization is now thought to be more complex and dependent on several factors. For example, the skin and any mucosal surface of the body are colonized directly after birth by a wide range of bacteria. These bacterial communities evolve into a complex ecosystem during the first years of life, varying greatly among individuals and over time [8], [9]. Similarly, the microbiome of the upper respiratory tract appears to be influenced by the host genetic background, age, and factors that determine environmental exposure, such as social status, antibiotic use, vaccination, season, smoking, and the pattern of social contacts, such as day care attendance or number of siblings [10], [11]. Furthermore, site-specific factors and characteristics of the microbe itself also play a role. By colonizing a niche, a microbe should be able to survive local clearance mechanisms (i.e., mucus, ciliae), attach to the epithelium, rely on locally available nutrients, and bypass surveillance by the host immune system. Another essential condition for colonization is to outcompete inhabitants that were already present in the upper respiratory tract [12], [13]. To this end, microbes have developed a range of different interaction tools that lead to both negative and positive interactions. Positive associations are assumed to exist when one microorganism generates a favorable condition for another via mutualism, commensalism, symbiosis, or by helping to evade the host immune system. Negative associations may be due to direct interspecies interactions (via ammensalism or predation), when organisms directly compete for the same niche, or when host immune responses disproportionally affect one of the competing microorganisms.

*S. pneumoniae*, *H. influenzae*, *M. catarrhalis*, and *S. aureus* are commonly recognized etiological agents in respiratory tract infections. However, colonization by these species is also very common under healthy circumstances, with high colonization rates in children in particular [10], [11], [14]–[17]. Since these frequent colonizers all share the nasopharynx as their natural niche, it is likely that these species interact with one another even during healthy states. Margolis and colleagues [12] demonstrated the existence of such interactions in vivo by introducing *H. influenzae* into the nasopharynx of neonatal rats that had or had not been pre-colonized by *S. pneumoniae*. The authors reported an increase in *H. influenzae* density when *S. pneumoniae* was present, suggesting synergism between these bacterial species. However, when these two species were inoculated in the reverse order, inhibition was observed, indicating competition between both species. This discrepancy was found to be both strain-specific and site-specific within the nasal cavity.

Besides interactions between potential pathogenic bacteria, there is currently also considerable interest in possible interactions between commensals and potential pathogenic microbes. Commensals are thought to play an important role in preventing respiratory and invasive disease. Possible mechanisms by which commensals might prevent disease are inhibition of colonization and expansion of potential pathogens, immune modulation, and stimulation of mucosal maturation and barrier function [5]. Most research on colonization resistance in the nasopharyngeal niche by commensals has been performed on alpha-haemolytic (AHS) and beta-haemolytic (BHS) streptococcal species [18]–[22]. An overview of the available evidence regarding interactions between pathobionts and between pathobionts and commensal bacteria can be found in Table S1 and Figure S1 in Text S1.

### Bacterial Mechanisms of Interaction

**Figure 1:**
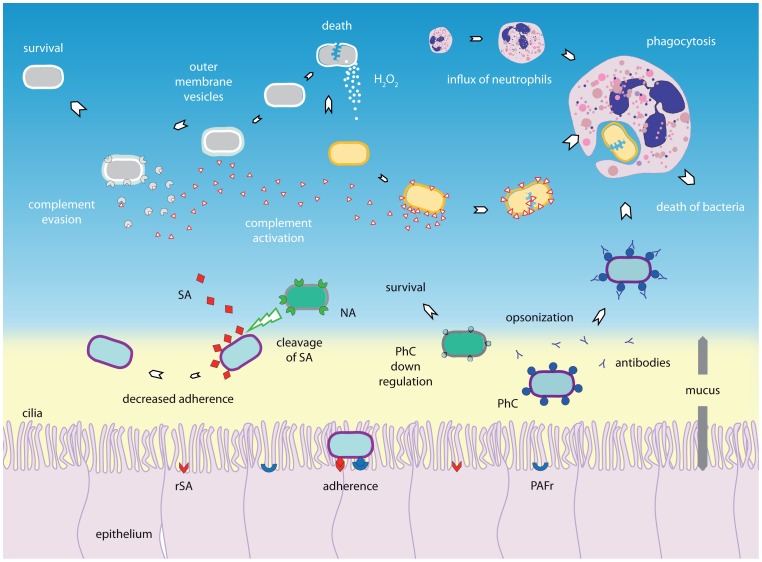
Bacterial–bacterial interactions. The composition of nasopharyngeal microbiota is constantly subject to interactions between species. Bacterial species can interact with other bacterial species by competition and synergism. Synergism can be characterized by, for instance, the production of components that favors another species, as shown for the production of outer membrane vesicles. These may contain factors that are able to inactivate complement factor C3, thereby allowing another species to escape the immune system. Production of substances by one species, for example hydrogen peroxide (H_2_O_2_), may eliminate its competitor. The immune system may also be involved in competition, as one bacterium has fewer escape mechanisms to evade the immune system than another and therefore may use co-inhabitants to survive, whereas the reverse phenomenon (i.e., one species may trigger the immune system to combat the other species) may also occur. In addition, since PhC (phosphorylcholine) is shown to be immunogenic and some species may be able to switch off PhC expression whereas others cannot, there might be a selective advantage. Another form of competition involves competition for the same host receptor, as demonstrated for PhC and its receptor platelet activating factor receptor (PAFr). Moreover, one species may use neuraminidase to cut off the sialic acids (SA) that other bacteria may require for attachment to host receptors, therefore inhibiting adherence of the other bacterial species. H_2_O_2_, hydrogen peroxide; PAFr, platelet activating factor receptor; PhC, phosphorylcholine; NA, neuraminidase; SA, sialic acid (SA); rSA, receptor for sialic acids; Ab, antibodies.

To date, several mechanisms have been proposed to explain bacterial–bacterial interactions observed in the upper respiratory tract. An overview of these mechanisms is illustrated in Figure 1.

One well-studied mechanism used by bacteria to compete with other species is the production of hydrogen peroxidase (H_2_O_2_), which is lethal for most bacteria. *S. pneumoniae* is exceptionally tolerant to H_2_O_2_ and produces concentrations that are bactericidal even for bacteria that produce the H_2_O_2_-neutralizing enzyme, catalase, such as *S. aureus* [23] and *H. influenzae* [24]. Genetically modified pneumococcal strains that are unable to produce H_2_O_2_ therefore also lose this ability to kill other strains [23], [24], demonstrating how reliant pneumococcal strains are on this system for survival. On the other hand, in vivo experiments with pneumococcus strains that do not produce H_2_O_2_ showed no impact on the survival of other species; however, since different strains were used in those studies, phenotypic differences could be responsible for discrepancies between in vitro and in vivo results [25], [26].

Another strategy used by competing species to interfere with each other is targeting structures that mediate adherence to the epithelium of the competing microorganism. For example, neuraminidase expressed by the pneumococcus is able to cut off cell surface–expressed sialic acids of some *H. influenzae* strains, thereby preventing attachment to the surface of airway cells and subsequent colonization [27].

A third, well-described interaction mechanism involves phosphorylcholine, a cell-surface molecule that mediates bacterial adherence to host cell receptors. Phosphorylcholine is expressed by both *S. pneumoniae* [28] and *H. influenzae* [29], and seems to contribute to the competitive effect between these two species through its immunogenicity [30]. Pre-exposure to one of the two species induces the production of antibodies against phosphorylcholine, thereby promoting clearance of the other co-colonizing species [30], [31]. Since in vitro studies have shown that phosphorylcholine is necessary for the survival of pneumococci but not *H. influenzae*, the latter may switch off phosphorylcholine expression to give it an advantage over *S. pneumoniae* [29], [31].

The host immune system is also involved in interspecies competition, as has been elegantly shown in vivo by Lysenko et al. [32]. When *S. pneumoniae* was co-colonized with an *H. influenzae* strain, the density of *S. pneumoniae* was lower than when inoculated alone, and this proved to be fully dependent on complement- and neutrophil-mediated killing of pneumococci [32], [33]. In addition to innate immunity, the components of the adaptive immune system may play a role in microbial interactions. This is supported by a large epidemiological study that reported a significant negative association between *S. pneumoniae* and *S. aureus* in HIV-uninfected, but not HIV-infected, children [34]. Furthermore, HIV infection has been associated with increased pneumococcal carriage rates compared with unaffected individuals. Therefore, it is suggested that a possible failure of the adaptive immune system, mainly CD4 T-cell-mediated [35] and decreased mucosal immunity [34], may contribute to the absence of a negative association between *S. pneumoniae* and *S. aureus* in immunocompromised HIV-infected hosts.

Alternatively, one bacterium can also promote the co-colonization of another bacterium, for example by inducing immune evasion, as has been described for *H. influenzae* and *M. catarrhalis*. *M. catarrhalis* is able to release outer membrane vesicles (“blebs”) containing ubiquitous surface proteins. Using different processes, these proteins are able to deactivate complement factor C3, which is a crucial amplifier of the complement system. *M. catarrhalis* may release these vesicles during co-colonization with *H. influenzae*, thereby protecting *H. influenzae* from complement-mediated killing [36]. A summary of evidence regarding bacterial–bacterial mechanisms occurring at the respiratory tract is given in Figure 1. It should be noted, however, that the presence of one bacterial strain may affect the outcome of competition between other bacteria [33], and therefore interaction patterns between species are probably far more complex than the “simple” interaction between two species. In addition, in vitro and in vivo studies revealed discrepancies in the presence and direction of interspecies interactions, for example in the interaction between *S. pneumoniae* and *H. influenzae* [24], [32], supporting an important role for host factors in the observed interspecies interactions.

In summary, it is plausible that microbial interactions are multifactorial and involve a complex interplay between multiple host factors and bacterial characteristics that may have important consequences for both the composition and stability of the microbial community itself and susceptibility to disease [37].

## Viral–Bacterial Interaction

Interactions between viruses and bacteria in the pathogenesis of respiratory infections have been extensively reported in the literature. Perhaps the most well-known viral–bacterial interaction is the synergism between influenza virus and *S. pneumoniae* [38]. Although an influenza virus infection alone can be fatal, mortality increases dramatically when a bacterial super-infection occurs, as in the case of the “Spanish flu” pandemic in 1918–1919 when millions of people died, most from secondary pneumococcal pneumonia [38]. This is further underlined by animal experiments showing that death occurred in 35% and 15% of mice infected with either influenza virus or pneumococcus, respectively, whereas 100% of mice infected with both pathogens simultaneously succumbed to infection within one day [39]. Besides synergism between influenza virus and *S. pneumoniae*, other interactions between viral and bacterial species have been described in the literature, as shown in Table 1 [39]–[69].

**Table 1:** Viral–bacterial interaction based on data available from human, animal, and in vitro studies.

Virus	Bacterium	Association	Human Studies Asymptomatic Children	Animal Studies	In Vitro Studies
				Murine	Type Epithelium
**Human rhinovirus**	*S. pneumoniae*	+	Healthy [67]	NA	Nasal [40]
					Airway [41]
	*H. influenzae*	+	Otitis-prone [68]	NA	Nasal [40]
					Primary airway [42]
					Bronchial [42], [43]
	*S. aureus*	+	NA	NA	Nasal [40]
					Alveolar basal [44]
	*M. catarrhalis*	+	Otitis-prone [68], [69]	NA	NA
**Human metapneumovirus**	*S. pneumoniae*	*+*	NA	Mice [54]	Bronchial [45]
**RSV**	*S. pneumoniae*	*+*	NA	Mice [46], [55]	Nasopharyngeal [46], [47]
					Bronchial [48]
					Small airway [48]
					Alveolar basal [46]–[49]
	*H.influenzae*	*+*	NA	Chinchillas [56]	Nasopharyngeal [50]
					Bronchial [48]
					Small airway [48]
					Alveolar basal [48], [51], [52]
**Influenza virus**	*S. pneumoniae*	*+*	NA	Mice [39], [54], [57]–[60]	Bronchial [48]
				Tracheal explants (ex vivo) [61]	Small airway [48]
					Alveolar basal [48]
	*H. influenzae*	*+*	NA	Mice [62]	Bronchial [48]
					Small airway [48]
					Alveolar basal [48]
	*S. aureus*	*+*	NA	Mice [63], [64]	NA
**Parainfluenza virus**	*S. pneumoniae*	*+*	NA	NA	Bronchial [48]
					Small airway [48]
					Alveolar basal [48]
	*M. catarrhalis*	*+*	Healthy [67]	NA	NA
**Adenovirus**	*S. pneumoniae*	*+*	NA	NA	Nasopharyngeal [53]
					Alveolar basal [53]
	*H. influenzae*	*+*	Otitis-prone [68]	Chinchilla [65]	NA
	*M. catarrhalis*	*+*	Healthy [68]	NA	NA
**Coronavirus**	*H. influenzae*	*+*	NA	Rats [66]	NA

Virus (column one) and respective bacterium (column two) for which interactions were observed (column three), and source of evidence: from human studies (column four), animal studies (column five), or in vitro studies (column six) showing type of epithelium tested.

NA, data not available from literature.

**Figure 2:**
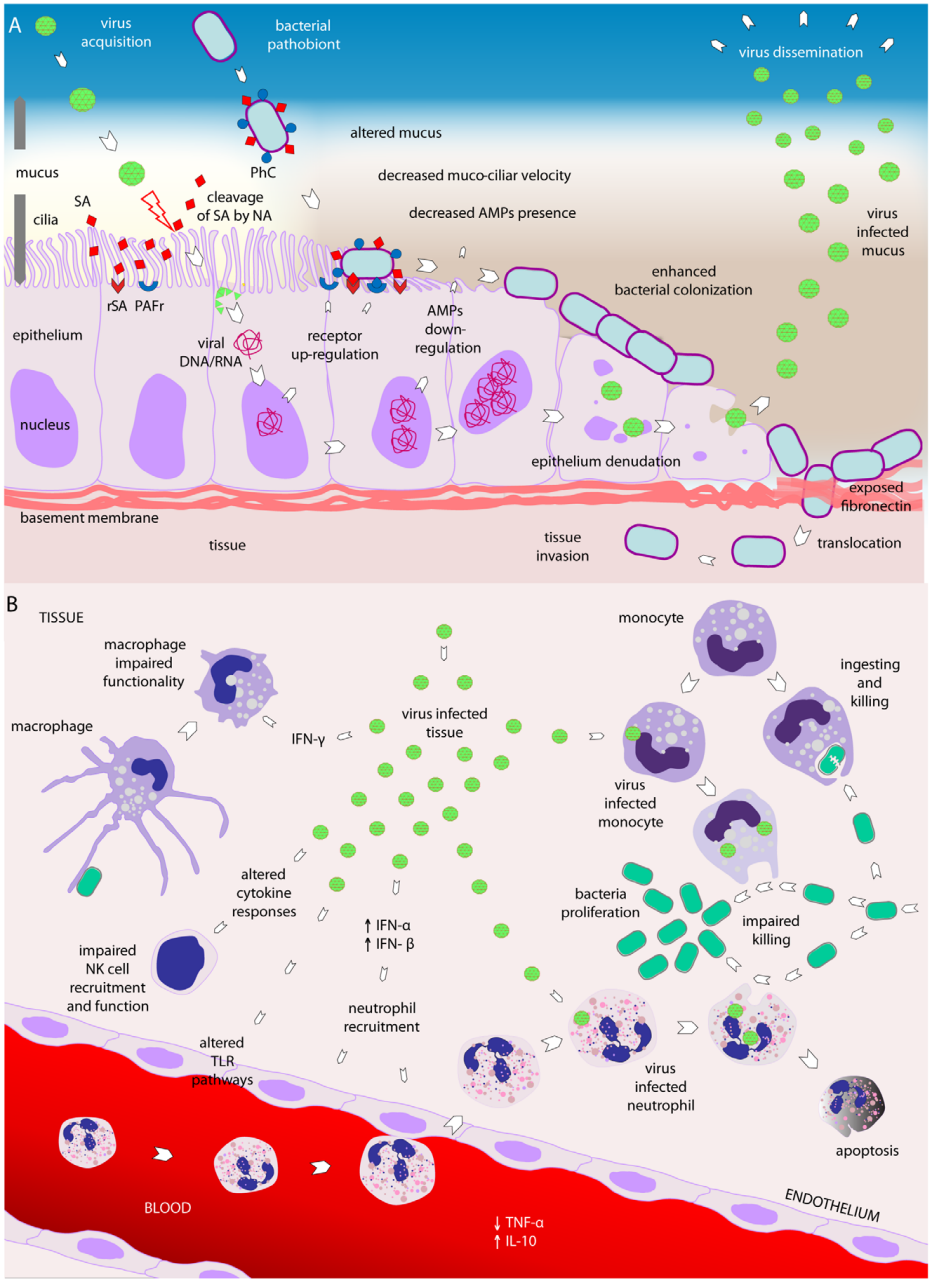
Viral–bacterial interactions. (A) Viral–bacterial interaction on the respiratory epithelial surface. Viral presence is thought to predispose the respiratory niche to bacterial colonization by different mechanisms. First, viruses may render the epithelium more susceptible to bacterial colonization by altering the mucosal surfaces. Ciliae may be damaged, leading to decreased mucociliar function of the respiratory epithelium. Additionally, due to viral-induced damage and loss of integrity of the epithelium layer, bacterial colonization may be enhanced and translocation may be increased. Virus-infected cells may decrease the expression of antimicrobial peptides, as shown for β-defensins, thereby affecting the natural defense of the host epithelium. Viral neuraminidase (NA) activity is able to cleave sialic acids residues, thereby giving access to bacterial receptors that were covered by these residues. Finally, viruses may induce bacterial colonization and replication both directly and indirectly, the latter by inducing upregulation of various receptors required for bacterial adherence, including PAFr, CAECAM-1, P5F, ICAM-1, and G-protein. PAFr, platelet activating factor receptor; ICAM-1, intracellular adhesion molecule 1; P5 fimbriae, outer membrane protein P5-homologous fimbriae; CAECAM-1, carcinoembryonic adhesion molecule-1; PhC, phosphorylcholine; SA, sialic acids; rSA, receptor for sialic acids; NA, neuraminidase; mRNA, messenger RNA, AMPs, antimicrobial peptides. (B) Viral–bacterial interaction in relation to the host immune system. Viruses may also induce changes in immune function favorable to bacterial invasion: fewer NK cells may be recruited into the tissue and their functionality may be suboptimal as a consequence of viral infection. Virus-induced IFN-α and IFN-β may impair recruitment and functionality of neutrophils, and subsequently induce apoptosis of neutrophils recruited to combat the viral invader. Furthermore, IFN-γ seems to negatively affect the activity of macrophages. Viral-infected monocytes appear less effective in ingesting and killing bacteria, predisposing them to bacterial overgrowth and invasion. Viral infection seems to impair TLR pathways, induce production of the anti-inflammatory cytokine IL-10, and decrease the concentration of the pro-inflammatory cytokine TNF-α, generally affecting adequate immune responses to bacterial infections. Black arrows indicate increased (↑) or decreased (↓) activity or functionality of a cytokine. IFN, interferon; TNF, tumor necrosis factor; TLR, toll like receptor; IL, interleukin; NK cell, natural killer cell.

The mechanisms by which viruses influence bacterial colonization and invasion are very diverse. We have summarized the known mechanisms in Figure 2a and 2b and will discuss each of these mechanisms below.

### Viral Predisposition to Bacterial Adherence

Since attachment of a pathogen to mucosal surfaces is the first step towards respiratory disease, and viral infection alters the defense of the host epithelium in general [70], it has been postulated that viral presence may render the epithelium more susceptible to bacterial colonization [10]. Mouse studies have shown that viral predisposition to bacterial attachment not only occurs in case of a simultaneous infection, but also up to a week after initial viral infection [46], [48], [55] or even after full recovery from influenza infection [71]. Moreover, Hakansson et al. [53] demonstrated that not all viral types and bacterial species and strains interact to the same extent; only pneumococcal strains with high adhesive capacity were able to adhere to human respiratory epithelium infected with adenovirus, and this effect was restricted to types of adenoviruses generally able to cause respiratory disease in humans.

### Disruption of the Epithelium Barrier

The epithelial layer of the respiratory tract mucosa is the first line of defense against a bacterial invader: loss of barrier function could therefore lead to entry of pathogens. Viruses generally replicate intracellularly and can subsequently disarrange cellular processes or kill infected cells through metabolic exhaustion or direct lysis. Induced cell death may in turn lead to denudation of the epithelial layer [59], [65], exposing the basement membrane. *S. pneumoniae* was found to bind strongly to fibronectin, which is prominently exposed at the basement membrane after denudation of epithelium [72]. Similarly, *S. aureus* [73] and *M. catarrhalis* [74] have been shown to bind to extracellular matrix proteins, suggesting that these species might also benefit from virus-induced damage to epithelium. Furthermore, the binding capacities of bacteria to fibronectin appear to be strongly influenced by the amount of exposed fibronectin and exposure duration [72], and since viral presence may directly induce upregulation of fibronectin expression, as has been shown for rhinovirus, this will additionally enhance pathobiont binding [40].

Another consequence of disrupted epithelium is the loss of epithelial integrity and decreased inhibition of bacterial translocation. This has been clearly shown for rhinovirus-induced paracellular migration of *H. influenzae* [75]. Viruses may also induce damage to ciliated cells, resulting in decreased mucociliar velocity and impaired bacterial clearance [61], [65].

### Upregulation of Adhesion Proteins

Viral presence in the infected cells may alter the expression of antimicrobial peptides, also known as defensins [76], secreted in the respiratory mucosa [56], which are key innate immune components that directly eliminate pathogenic bacteria [76]. Viral infection also triggers a pro-inflammatory response that leads to upregulation of adhesion proteins in a range of cells, including epithelial cells. These adhesion proteins act as receptors that allow immune cells to bind to virus-infected cells and combat the viral invader. This is illustrated by the upregulation of eukaryotic cell surface receptors such as intracellular adhesion molecule 1 (ICAM-1), outer membrane protein P5-homologous fimbriae (P5 fimbriae), carcinoembryonic adhesion molecule-1 (CEACAM-1), and platelet-activating factor receptor (PAFr) in different cell types upon infection with a virus such as respiratory syncytial virus (RSV) or para-influenza virus [48], [52]. Several bacterial species are able to adhere to a diverse group of these adhesion proteins on the surface of host cells [28], [40], [41], [48], [77]. For example, rhinovirus is able to induce upregulation of ICAM-1 needed for its own invasion as well as for adhesion of *H. influenzae* [43], [48]. Moreover, some strains of *S. pneumoniae* and *H. influenzae* express the natural PAFr-ligand phosphorylcholine, which also allows them to attach to, and invade, host cells. Increased PAFr expression in reaction to a viral infection may therefore facilitate adherence of both *S. pneumoniae* and *H. influenzae* [40], [41], [48]. However, influenza viruses might form an exception, as in vitro studies have found that influenza virus did not alter the expression of several receptors, including ICAM-, CAECAM, and PAFr [48]. In particular, conflicting data have been reported for a potential role of PAFr in the protection against influenza-related bacterial superinfection in mouse models [39], [78], though this might be explained by strain-related differences as well as the timing and order of viral and bacterial exposure [78].

### Production of Viral Factors

Influenza virus is thought to increase bacterial adherence by alternative mechanisms, such as the ability to produce neuraminidase (NA). NA produced by influenza and para-influenza viruses creates an entry point for bacteria into host cells by cleaving sialic acids residues, thereby exposing bacterial receptors on the surface of the upper respiratory tract [79]–[81]. This is supported by several in vitro and animal studies, including studies on the effects of NA inhibitors [58], [81], [82]. Although some bacteria such as *S. pneumoniae* naturally express NA [83], the contribution of bacterial NA to viral replication seems to be negligible, most likely due to poor enzymatic activity and stringent binding requirements of bacterial NA compared to viral NA [84].

RSV, on the other hand, does not produce NA. Instead, adherence of bacteria to RSV-infected cells is thought to be directly mediated by expression of RSV-protein G [46], [47], [51]. Blockade of G-protein activity, however, does not completely reduce excess bacterial colonization in RSV-infected cells in vitro [51]. This implies that other mechanisms might be involved during viral–bacterial co-occurrence, such as upregulation of additional receptors like ICAM-1 and PAFr [48], [51] or other indirect pathways.

### Dysfunction of Immune System Components

As described above, viral-induced expression of adhesion molecules may support adhesion of neutrophils, monocytes, and other immune cells to virus-infected cells. This may increase recruitment and activation of pro-inflammatory immune cells. However, respiratory viruses may also directly affect the immune system, for example by impairment of neutrophil function, decreased oxidative burst [55], [85], and enhanced neutrophil apoptosis, thereby increasing susceptibility to bacterial superinfection [85]–[87]. Additionally, some strains of influenza virus infection may predispose to superinfection by *S. aureus* due to ineffective natural killer (NK) cell recruitment and activation [64]. Viral infection may also alter monocyte function, resulting in lower surface expression of CD receptors [50]. In addition, viral presence also affects the production and biological activity of cytokines [54]. For example, virus-induced interferon (IFN)-α and IFN-β induce impaired neutrophils responses due to inadequate production of neutrophil chemoattractants [88]. In addition, IFN-γ downregulates the activity of macrophages [89], thus impairing bacterial clearance in its initial phase. It has also been shown that blockage of IFN-γ decreases susceptibility to secondary bacterial pneumonia in mice [89]. Moreover, tumor necrosis factor (TNF)-α production is downregulated during viral infection, which may also lead to increased susceptibility to secondary bacterial infections [50]. Respiratory viruses can also interact with toll-like receptor (TLR) pathways, preventing appropriate routing of immune responses [90]. This is, for example, illustrated by data obtained from a co-infection model with influenza virus and *S. pneumoniae* in mice, where excessive immunosuppressive interleukin (IL)-10 production following co-infection has been observed, which was associated with enhanced bacterial colonization and increased mortality [71].

### Unidirectional or Bidirectional Synergism

Most studies point towards a unidirectional viral predisposition to bacterial colonization. However, there are some clues that a preceding bacterial infection may also increase susceptibility to a consecutive viral infection. For example, Sajjan et al. [42] showed that *H. influenzae* is able to stimulate expression of ICAM-1 and TLR-3 on human airway epithelial cells, providing an entry point for rhinovirus. Another report suggested that human bronchial epithelial cells pre-incubated with pneumococcus, but not with *H. influenzae*, *M. catarrhalis*, or *S. aureus*, were more susceptible to human metapneumovirus [45]. Moreover, it might also be possible that microbial interactions may disturb the equilibrium of the microbiota, creating an opportunity for viral invasion and transmission. This was recently underlined by Kuss et al. [91], who showed that transmission of an enteric virus was less successful when the intestinal microbiota of mice were disbalanced by antibiotic treatment. Importantly, viruses might even be capable of using their microbial environment to escape immune clearance [92]. Little information exists, however, regarding bacterial predisposition to viral disease, and further research is needed to unravel the extent to which bacteria enhance viral presence.

## Asymptomatic Presence of Viruses In Vivo

The impact of viral presence could be far more extensive than currently thought. In addition to bacterial commensals, viruses are also commonly found in the nasopharynx of asymptomatic individuals. With the introduction of viral PCR techniques, it has become feasible to detect and distinguish between respiratory viruses in larger epidemiological studies. A concise review showed that up to 68% of respiratory samples from asymptomatic individuals were positive for respiratory viruses [93]. When specifying these numbers for symptom-free children, studies have reported presence rates of 16%–33% in developed communities [68], [94]–[96] and 4%–52% in developing communities [68], [97]–[100]. Interestingly, children in some native populations, such as Australian Aboriginals and Alaska Yup'ik Eskimos, are known to be more susceptible to diseases caused by respiratory pathogens, and also seem to more frequently carry respiratory viruses during healthy periods [68], [100]. A detailed overview of data on the asymptomatic presence of viruses is presented in Table 2.

**Table 2:** Viral detection in respiratory samples in asymptomatic children.

Year	Season^a^	Number^b^	Age	Risk Group	Viral Findings									
					*n* (%)									
					Picorna		AdV	HBoV	RSV	hMPV	CoV	IV	PIV	Polyoma Viruses
					HRV	EV								
2011 [67]	Autumn, winter, spring	66	6 m–3 y	Healthy	28 (42%)	5 (7.6%)	4 (6.1%)	13 (20%)	6 (9.1%)	1 (1.5%)	5 (6.1%)	0 (0%)	6 (9.1%)	WU 9 (14%); KI 1 (1.5%)
2011 [99]	All year	34	<1 y	Healthy	8 (24%)									
		51	1–4 y	Healthy	7 (14%)									
		69	5–19 y	Healthy	9 (13%)									
2011 [95]	Winter	30	<1 y	Healthy	6 (18%)	0 (0%)	1 (3%)	3 (9%)	2 (6%)	0 (0%)	6 (18%)	2 (6%)	0 (0%)	
		23	1–2 y	Healthy	4 (16%)	0 (0%)	0 (0%)	0 (0%)	0 (0%)	0 (0%)	2 (8%)	0 (0%)	0 (0%)	
		97	2–6 y	Healthy	14 (15%)	1 (1%)	0 (0%)	2 (2%)	1 (1%)	0 (0%)	3 (3%)	3 (3%)	0 (0%)	
2010 [68]	All year	570	<2 y	Healthy	94 (17%)		20 (3.5%)		3 (0.5%)	0 (0%)	8 (3.6%)	3 (0.6%)	4 (1.8%)	
		436	<2 y	At risk	103 (24%)		37 (8.5%)		2 (0.5%)	3 (1.8%)	6 (3.5%)	2 (0.5%)	3 (1.8%)	
2010 [98]	Autumn, winter	272	<3 y	Rural					3 (1.1%)	1 (0.4%)		2 (0.7%)	5 (1.8%)	
2010 [97]	All year	57	<12 y	Rural					2 (4%)					
2010 [100]	All year	425	<3 y	At risk	140 (33%)		68 (16%)		18 (4%)	29 (7%)	15 (4%)	3 (1%)	13 (3%)	
2009 [101]	Autumn, winter, spring	65	<7 y	Healthy	14 (22%)	2 (3%)	0 (0%)		0 (0%)	0 (0%)	5 (8%)	0 (0%)		
2008 [102]	All year	116	<14 y	Healthy	11 (9.5%)		5 (4.3%)	2 (1.7%)	1 (0.8%)	1 (0·8%)	0 (0%)	0 (0%)	0 (0%)	
2008 [112]	Autumn, winter, spring	100	≤3 y	Healthy				43 (43%)						
2007 [103]	Autumn, winter, spring	269	1.5–9.3 y	Healthy	29 (11%)				2 (1%)		2 (1%)	1 (0%)	1 (0%)	
2006 [96]	All year	456	<1 y	High risk of atopy	52 (11%)		2 (0%)		24 (5%)	1 (0%)	20 (4%)	0 (0%)	4 (1%)	
2006 [104]	All year^c^	410	1–9 y	Healthy	37 (9%)^d^									
2004 [94]	NS	70	5 m	Healthy	12 (17%)				2 (3%)		3 (4%)	1 (1%)	1 (1%)	
		64	1 y	Healthy	18 (28%)				0 (0%)		0 (0%)	0 (0%)	0 (0%)	
		38	1.5 y	Healthy	10 (26%)				0 (0%)		0 (0%)	0 (0%)	0 (0%)	
		49	2 y	Healthy	7 (14%)				0 (0%)		1 (2%)	0 (0%)	0 (0%)	

a Related to geographical area.

b Number of samples tested.

c Stratified for season.

d Picornavirus general.

M, months of age; Y, years of age; HRV, human rhinoviruses; EV, entero viruses; AdV, adeno viruses; HBoV, human bocavirus; RSV, respiratory syncytial virus; hMPV, human metapneumovirus; CoV, corona viruses; IV, influenza viruses; PIV, para-influenza viruses; NS, not specified.

Differences between studies are likely to be explained by inclusion criteria and heterogeneity of populations due to differences in age, sample size, genetic background, season of sampling, lifestyle, and environmental circumstances, as well as health status and registration of respiratory symptoms prior to or following sampling.

The interpretation of viral presence in human respiratory samples is therefore becoming increasingly complex. In children, Singleton et al. [100] proposed dividing respiratory viruses into two groups, depending on their viral contribution to disease. The contributing factor to illness of a given viral pathogen was estimated by the proportion of all hospitalized cases related to this virus divided by its presence rate in asymptomatic children. Group 1 includes viruses with a significantly greater contribution to respiratory symptoms, including RSV, metapneumovirus, certain para-influenza viruses, and influenza viruses. Group 2 viruses, including human rhinoviruses, adenoviruses, and coronaviruses, are less likely to be the single causative pathogen of disease in children. The exact role of these group 2 viruses in the pathogenesis of respiratory infections remains unanswered, but it seems plausible that they might have a more subtle or indirect role in the pathogenesis of respiratory infections. In general, however, presence rates of viruses are higher in symptomatic individuals compared to asymptomatic individuals. Moreover, most of these viruses are associated with an increased presence or density of bacterial pathogens, supporting a role for overgrowth of, and/or invasion by, pathogenic bacteria and, consequently, the development of respiratory infections.

Interestingly, it has also been shown that up to 27% of asymptomatic healthy children carry multiple respiratory viruses in their nasopharynx at any given time [68], [94], [95], [97], [99]–[103]. For example, RSV-positive samples were also positive for rhinovirus [95], [103] or bocavirus [67]. Additionally, co-incidence of multiple “innocuous” viruses was frequently observed, such as co-occurrence of rhinovirus with adenovirus [68], [99], [102], [103] or coronavirus [95], [103] in children without respiratory symptoms. So far, study sizes have been too small to determine whether viral co-occurrence in asymptomatic children is an accidental or season-related event, or, alternatively, whether the presence of virus A predisposes to the acquisition of virus B. New studies are needed to elucidate the possibility of true synergism between viruses and the extent to which this contributes to the pathogenesis of respiratory infections.

The asymptomatic presence of viruses in the nasopharynx may be explained by several mechanisms [93], [95]. First, one cannot rule out the possibility that the PCR detection of the virus preceded a symptomatic episode, i.e., viral presence was observed during the incubation period [94], [104]. Second, in studies that involve young children, parental registration of the infant health status might be a major confounder, as the presence of minor respiratory symptoms like a runny nose may be underestimated. Third, viral presence might indicate a true subclinical infection. A recent study [95] revealed that the median viral load of rhinovirus was significantly lower in asymptomatic children than in symptomatic children, with a total absence of clinical symptoms when the viral load was below a certain threshold. Another study analyzed the bronchoalveolar lavage fluids of asymptomatic children attending elective surgery to reveal the effect of viral presence on the immune system. The authors found that viral presence was associated with significantly higher neutrophil counts, but not macrophage, lymphocyte, and eosinophil counts [105]. This may imply that a low viral load only triggers a minor inflammatory response without causing respiratory symptoms. Fourth, the duration of viral shedding varies greatly between studies and seems to be strongly dependant on viral species, selected patient population, and method of detection [104], [106]–[108]. Therefore, detected viruses may mark an expiring infection. Finally, prolonged detection of viral presence may be due to the sequential presence of different serotypes of the same viral species [104], [109]. For example, for adenovirus, it was recently shown that prolonged or repeated persistence of viral nucleic acids might actually be caused by both persistent viral shedding and consecutive infection with different serotypes/strains [108]. However, few other studies discriminate between the exact serotypes of the viruses found in the nasopharynx of asymptomatic children [99]. Most likely, interplay of these factors will influence the presence of viral species in the nasopharynx of healthy asymptomatic children, though the clinical relevance of these findings remains unclear and needs further investigation.

## Viral–Bacterial Interaction in Asymptomatic Humans

It is becoming clear that viruses present in the nasopharynx of asymptomatic individuals can facilitate both colonization of bacteria and further viral presence. For example, several cohort studies of asymptomatic children have found a positive correlation between the presence of adenovirus and rhinovirus and both *M. catarrhalis* and *H. influenzae* (see Table 1 for summary of these and other studies) [68], [69]. However, due to the cross-sectional design of most of these studies, it remains unclear whether this reflects a true cause-effect relationship, and if so, in what direction these effects occur. To unravel the sequence of the observed effects, longitudinal studies with intensive follow-up during health and disease are needed.

## Model for Interspecies Interaction

**Figure 3:**
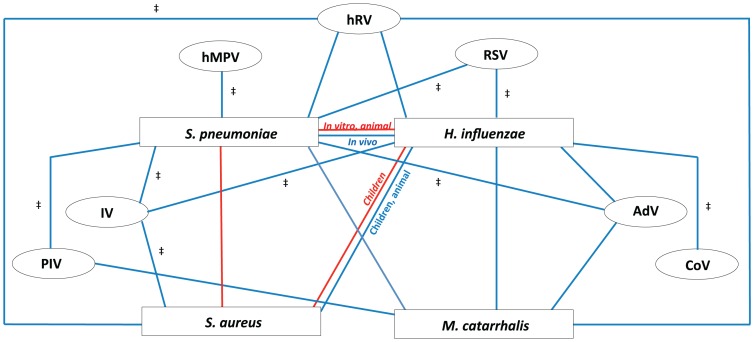
Proposed model of bacterial and viral interactions. This model represents the cumulative dynamics of bacterial and viral interactions occurring within the nasopharyngeal niche during asymptomatic episodes as observed in all cumulative literature references. All available information on the four main potential pathogenic bacteria (*Streptococcus pneumoniae*, *Haemophilus influenzae*, *Staphylococcus aureus*, and *Moraxella catarrhalis*) and seven common respiratory viruses (rhinoviruses (hRV), respiratory syncytial virus (RSV), adenoviruses (AdV), coronavirus (CoV), influenza viruses (IV), para-influenza viruses (PIV), and human metapneumovirus (hMPV)) are depicted. Red lines represent a negative association of co-colonization (competition), blue lines represent a positive association of co-colonization (synergism). For all depicted associations, evidence is available from human (surveillance) studies, except for those indicated with ‡, where evidence is only available from in vitro and/or animal studies.

In this review, we have synthesized as much knowledge as possible on interspecies interaction between potential pathogenic agents as well as non-pathogenic commensals. We have described different mechanisms by which these interactions may be facilitated, including direct bacterial effectors, viral-induced bacterial adhesion, viral-derived disruption of the epithelium, production of viral products, and interference with the host immune system. We have incorporated all available knowledge on in vitro research, animal experiments, and human data into a single theoretical model of interspecies interplay (Figure 3). The majority of data available on microbial interactions has been collected from experimental setting and epidemiological surveillances of combinations of a limited number of microorganisms. Recently, we described the extreme complexity of the microbial population in the upper respiratory niche, with high diversity of bacteria and high variability between individuals [110]. Moreover, Pettigrew et al. showed that nasal microbiome communities differ according to the health status of young children (i.e., healthy or presence of acute respiratory symptoms) [111], although due to the cross-sectional approach, it remains unclear whether this reflects a true cause-effect relation and in which direction this may occur. In addition to a bacterial microbiome, the presence of a diverse community of viruses (or viriome) in the upper respiratory niche may further increase the complexity of interactions within this ecosystem. We have attempted to accommodate these intricate interferences in our model. Ultimately, these interactions may strongly influence the dynamics within the complete microbial population of the respiratory niche and may lead to an imbalanced state with potential overgrowth of pathogens and progression towards consecutive disease. In particular, a role for viral co-infection in the observed dynamics within this microbiome deserves further investigation; viruses and microbiota may each influence the pathogenicity and consecutive development of infections of the other, as has recently been suggested for gut microbiota [91], [92].

Despite some discrepancies between in vivo and in vitro data, our model does provide a better understanding of the complex interspecies interactions within the respiratory niche. Inconsistencies between in vitro and in vivo studies, as well as between different study populations, underline the involvement of additional factors such as host immunity, genetic background, the commensal environment, available nutrients, and environmental circumstances.

For a better understanding of the mechanisms underlying the positive and negative interactions observed among species of the nasopharyngeal niche, intensive in vitro and in vivo research as well as longitudinal epidemiological studies using advanced data analyses are warranted. Special interest should be addressed to bacterial carriage and viral presence in asymptomatic children, for the upper respiratory niche may also function as an important reservoir of potential pathogenic bacteria and viral species in the community. New and more detailed knowledge regarding this complex interplay may help us to reconsider how we define the causative mechanisms of respiratory diseases.

In conclusion, this review summarizes the current knowledge on the mechanisms underlying bacterial and viral interactions in the respiratory tract. Although colonization of both respiratory bacteria and viruses is mostly asymptomatic, synergistic and competitive interspecies interactions appear to occur, potentially influencing and disturbing the natural equilibrium of the complex microbiota at the nasopharyngeal niche. We propose a multidimensional interaction model that underlines the complexity of interactions between potential pathogenic bacteria and respiratory viruses. Completing this model of interspecies interaction in the future will provide a better understanding of the dynamics of the complete respiratory ecosystem and may provide us with new insights into the potential role of an imbalanced equilibrium in the pathogenesis of respiratory disease—possibly the true key to disease.

**Search Strategy and Selection Criteria**

Relevant studies for this review were identified by searching PubMed and the reference lists of selected articles. Only articles published in English were included. We screened titles and abstracts on relevance: if relevant, we included the article in the construction of this review. Because we were specifically focusing on asymptomatic carriage of respiratory pathogenic species in children, we excluded studies based on symptomatic children, adults, and antimicrobial studies.

We searched for papers studying the four most important bacterial pathogens of respiratory tract infections known to interact with other microorganisms and viruses, namely *S. pneumoniae*, *H. influenzae*, *S. aureus*, and *M. catarrhalis* (search terms: “pneumococ*”, “Streptococcus pneumoniae”, “s. pneumoni*”, “Haemophilus influenzae”, “H. influenzae”, “Hemophilus influenzae”, “Staphylococcus aureus”, “S. aureus”, “staphylococ*”, *“Moraxella catarrhalis”, “M. catarrhalis”, “Moraxella catarrhalis”*). Respiratory viruses were defined by the following criteria (search terms: “adenovirus”, “adeno virus”, “boca”, “bocavirus”, “WU”, “Wupolyomavirus”, “WU-polyomavirus”, “KY”, “ky-polyomavirus”, “ky polyomavirus”, “influenza virus”, “influenza”, ”influenzavirus”, “parainfluenza virus”, para-influenzavirus”, “para influenza virus”, “corona-virus”, “coronavirus”, “corona virus”, “enterovirus”, “entero virus”, “parechovirus”, “parecho virus”, “RSV”, “respiratory syncytial virus”, “metapneumovirus”, “meta-pneumovirus”, “meta pneumovirus”, “rhinovirus”, “rhino virus”).

For the bacterial interactions, we used the search terms for the four bacteria of interest individually and combined. We combined these terms with the following search terms “interaction”, “co-exist*”, “interference”, “co-occurrence”, ”co-colonisation”, “synergism”, “antagonism”, “bactericidal”, “correlation”. We also performed a global search for mechanisms by which interactions may occur and searched in more detail for hydrogen peroxidase, phosphorylcholine, neuraminidase, and the host immune system.

With a focus on viral–bacterial interactions, we performed searches with the search terms for the four bacteria and 13 viruses described above. We combined different search terms to create a complete overview.

For studies on the asymptomatic presence of viruses, we performed a search with the viral search terms described above and combined them with “asymptomat*”, “without symptoms”, “health*”, “child*”, “infant*”, “human”.

To our knowledge, we have considered all relevant studies in the present review. However, when extensive literature was available, we decided to refer to a limited number of representative papers based on relevance, study size, and study design.

## Supporting Information

Text S1 Supporting information, including Table S1 (Bacterial–bacterial interaction) and Figure S1 (Proposed model of bacterial interactions at the upper respiratory tract).
(DOC)
