# Supplementary material for: Viral and Bacterial Interactions in the Upper Respiratory Tract
Source: PLoS Pathog. 2013 Jan 10;9(1):e1003057. doi: 10.1371/journal.ppat.1003057 (PMC3542149; doi:10.1371/journal.ppat.1003057)
Supplement: Text S1 — Supporting information, including Table S1 (Bacterial–bacterial interaction) and Figure S1 (Proposed model of bacterial interactions at the upper respiratory tract). (DOC) [file ppat.1003057.s001.doc]

**Text S1**

**Table S1 Bacterial-Bacterial interaction**

| **Bacterium** | **Association** | **Bacterium** |  | **Reference** |
| --- | --- | --- | --- | --- |
| ***S. pneumoniae*** | + | *S. pneumoniae* | Rats | 12 |
|  | + | *H. influenzae* | Healthy Gambian children | 113 |
|  |  |  | Healthy Belgian children | 114 |
|  |  |  | Healthy Aboriginal children | 115 |
|  |  |  | Healthy Australian children | 116 |
|  |  |  | Healthy South African children | 16 |
|  |  |  | HIV positive South African children | 16 § |
|  |  |  | Rats | 12 |
|  |  |  | Mice | 117 |
|  | - | *H. influenzae* | Mice | 34 |
|  |  |  | Selective media (BHI agar) | 25 |
|  | + | *M. catarrhalis* | Healthy Aboriginal children | 115 |
|  |  |  | Healthy Aboriginal children | 116 |
|  |  |  | Healthy Australian children | 116 |
|  |  |  | Selective medium (BHI agar) | 25 |
|  | - | *S. aureus* | Healthy Venezuelan children | 118 |
|  |  |  | Healthy Taiwanese children | 119 |
|  |  |  | Healthy Dutch children | 17 |
|  |  |  | Healthy South African children | 16 |
|  |  |  | Healthy Dutch children | 15‡ |
|  |  |  | Healthy Israeli children | 14‡ |
|  |  |  | Selective medium (BHI agar) | 24 |
| ***H. influenzae*** | + | *H. influenzae* | Rats | 12 |
|  | + | *M. catarrhalis* | Healthy Belgian children | 114 |
|  |  |  | Healthy Dutch children | 120 |
|  |  |  | Healthy Aboriginal children | 116 |
|  |  |  | Healthy Australian children | 116 |
|  |  |  | Clinical specimens (human serum) | 37 |
|  | + | *S. aureus* | Healthy Belgian children | 114 |
|  |  |  | Rats | 12 |
|  | - | *S. aureus* | Healthy South African children | 16 |
| ***M. catarrhalis*** | - | *S. aureus* | Healthy Australian children | 116 |
| ***S. aureus*** | - | *S. aureus* | Mice | 121 |
|  |  |  | Rats | 12 |
|  |  |  | Alveolar basal epithelium | 121 |
|  |  |  | Nasal epithelium | 121 |
| **Group A β-haemolytic streptococci** | + | *M. catarrhalis* | Healthy American children  Alveolar basal epithelium | 18  19 |
| **Group A α-haemolytic streptococci** | - | *S. pneumoniae* | Healthy Swedish children  Selective media  Selective media | 20  21  22 |
|  | - | *H. influenzae* | Healthy Swedish children  Selective media  Selective media | 20  21  22 |
|  | - | *M. catarrhalis* | Healthy Swedish children  Selective media  Selective media | 20  21  22 |

Table S1 Bacterial-Bacterial interaction

Bacteria (column one) can interact (column two) with other bacteria (column three), as has been described in human, animal, and *in vitro* studies (column four), and its reference (column five). § *Only non-vaccine type S. pneumoniae;* ‡ *Only vaccine type S. pneumoniae*

**Figure S1**


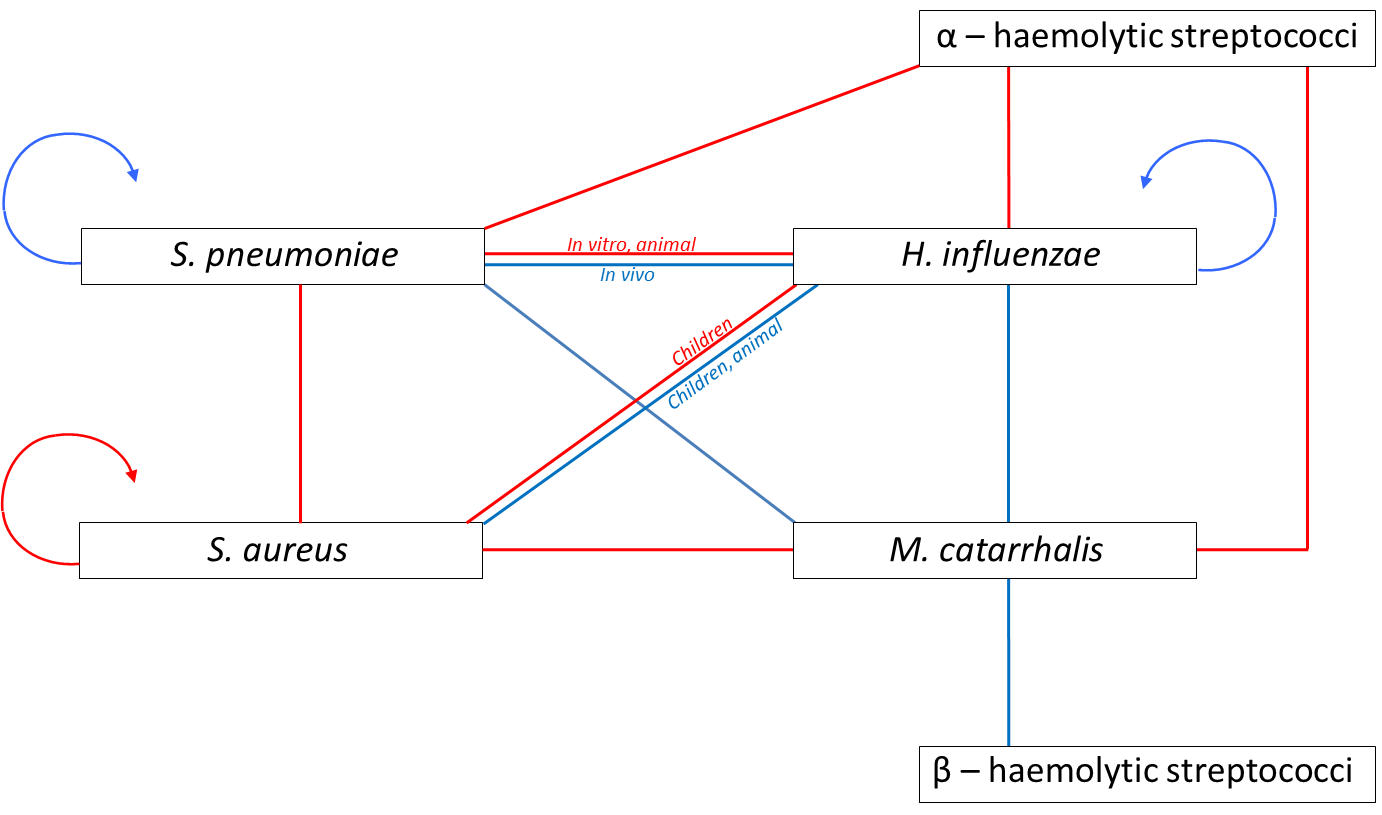


Figure S1 Proposed model of bacterial interactions at the upper respiratory tract.

Schematic graphical representation of interference in the nasopharyngeal niche between the four main potential pathogenic bacteria (*Streptococcus pneumoniae, Haemophilus influenzae, Staphylococcus aureus, and Moraxella catarrhalis*) and two groups of commensal bacteria (α-and β- haemolytic streptococci). Red lines represent a negative association of co-colonization (competition), blue lines represent a positive association of co-colonization (synergism).

Additional references

113. Jacoby P, Watson K, Bowman J, Taylor A, Riley TV, et al. (2007) Modelling the co-occurrence of Streptococcus pneumoniae with other bacterial and viral pathogens in the upper respiratory tract. Vaccine 25(13): 2458-64. 10.1016/j.vaccine.2006.09.020.

114. Lim J, Ha U, Sakai A, Woo C, Kweon S, et al. (2008) Streptococcus pneumoniae synergizes with nontypeable Haemophilus influenzae to induce inflammation via upregulating TLR2. BMC Immunology 9(1): 40.

115. Quintero B, Araque M, van der Gaast-de Jongh, C., Escalona F, Correa M, et al.
Epidemiology of Streptococcus pneumoniae and Staphylococcus aureus colonization in healthy Venezuelan children. Eur J Clin Microbiol Infect Dis 30(1): 7-19.

116. Kuo C, Hwang K, Hsieh Y, Cheng C, Huang F, et al. (2011) Nasopharyngeal carriage of Streptococcus pneumoniae in Taiwan before and after the introduction of a conjugate vaccine. Vaccine 29(32): 5171-7. 10.1016/j.vaccine.2011.05.034.

117. van Gils E, Hak E, Veenhoven R, Rodenburg G, Bogaert D, et al. (2011) Effect of seven-valent pneumococcal conjugate vaccine on Staphylococcus aureus colonisation in a randomised controlled trial. PLoS ONE 6(6): e20229.

118. Bogaert D, van Belkum A, Sluijter M, Luijendijk A, de Groot R, et al. (2004) Colonisation by Streptococcus pneumoniae and Staphylococcus aureus in healthy children. The Lancet 363(9424): 1871-72. DOI: 10.1016/S0140-6736(04)16357-5.

119. Regev-Yochay G, Dagan R, Raz M, Carmeli Y, Shainberg B, et al. (2004) Association between carriage of Streptococcus pneumoniae and Staphylococcus aureus in children. JAMA 292(6): 716-20.

120. Verhaegh SJC, Snippe ML, Levy F, Verbrugh HA, Jaddoe VWV, et al. (2011) Colonization of healthy children by Moraxella catarrhalis is characterized by genotype heterogeneity, virulence gene diversity and co-colonization with Haemophilus influenzae Microbiology 157: 169-78.

121. Barbagelata MS, Alvarez L, Gordiola M, Tuchscherr L, von Eiff C, et al. (2011) Auxotrophic mutant of Staphylococcus aureus interferes with nasal colonization by the wild type. Microb Infect 13(12-13): 1081-90. 10.1016/j.micinf.2011.06.010.
